# Genetic and Genomic Analysis of *Rhizoctonia solani* Interactions with Arabidopsis; Evidence of Resistance Mediated through NADPH Oxidases

**DOI:** 10.1371/journal.pone.0056814

**Published:** 2013-02-25

**Authors:** Rhonda C. Foley, Cynthia A. Gleason, Jonathan P. Anderson, Thorsten Hamann, Karam B. Singh

**Affiliations:** 1 CSIRO Plant Industry, Centre for Environment and Life Sciences, Perth, Western Australia, Australia; 2 The University of Western Australia Institute of Agriculture, University of Western Australia, Perth, Western Australia, Australia; 3 Division of Biology, Imperial College London, London, United Kingdom; National Taiwan University, Taiwan

## Abstract

*Rhizoctonia solani* is an important soil-borne necrotrophic fungal pathogen, with a broad host range and little effective resistance in crop plants. Arabidopsis is resistant to *R. solani* AG8 but susceptible to *R. solani* AG2-1. A screen of 36 Arabidopsis ecotypes and mutants affected in the auxin, camalexin, salicylic acid, abscisic acid and ethylene/jasmonic acid pathways did not reveal any variation in response to *R. solani* and demonstrated that resistance to AG8 was independent of these defense pathways. The Arabidopsis Affymetrix ATH1 Genome array was used to assess global gene expression changes in plants infected with AG8 and AG2-1 at seven days post-infection. While there was considerable overlap in the response, some gene families were differentially affected by AG8 or AG2-1 and included those involved in oxidative stress, cell wall associated proteins, transcription factors and heat shock protein genes. Since a substantial proportion of the gene expression changes were associated with oxidative stress responses, we analysed the role of NADPH oxidases in resistance. While single NADPH oxidase mutants had no effect, a NADPH oxidase double mutant *atrbohf atrbohd* resulted in an almost complete loss of resistance to AG8, suggesting that reactive oxidative species play an important role in Arabidopsis's resistance to *R. solani*.

## Introduction

Soil borne pathogens cause heavy crop loses throughout the world. An important class of soil borne diseases are those caused by fungal pathogens. Broadly speaking there are two major classes of fungal pathogens, those with a biotrophic lifestyle and those with a necrotrophic lifestyle although some pathogens are hemibiotrophs that switch from a biotrophic phase to a necrotrophic phase [1]. Over the last two decades there has been much progress in our understanding of plant resistance to fungal pathogens, in part through the development and analysis of fungal pathosystems in the model plant, *Arabidopsis thaliana* (L) Heynh. While progress in understanding plant resistance has been greatest for fungal pathogens with a biotrophic or hemibiotrophic lifestyle, recently good progress has also been made on necrotrophs [2], [3]. In Arabidopsis, systems to study necrotrophs including *Botrytis cinerea* and *Alternaria brassicicola* have been developed and these are helping to identify genes and signalling pathways important for defence against these foliar pathogens [4], [5], [6], [7].

While good progress has been made in our understanding of plant defence against numerous foliar pathogens, an area where less progress has been made is with soil-borne pathogens which can differ significantly from foliar pathogens in terms of their ecology, lifecycle and infection strategies [8]. Perhaps the best studied soil-borne pathogen is the oomycete pathogen, *Phytophthora sojae* [9]. *Rhizoctonia solani* (Kühn) is a soil-borne fungus which causes disease on many economically important crops throughout the world [10] and belongs to the large genus *Rhizoctonia* that varies widely in morphology, ecology and pathology [11]. *R. solani* has been characterised and grouped into 13 anastomosis groups (AG) that vary in pathogenicity, physical characteristics and sequence variations [12], [13]. *R. solani* is well known for causing rice sheath blight which is one of the most prevalent rice diseases [14]. Isolates of AG8 cause bare patch disease of cereals and legumes [15], and can cause severe root rot in canola [16]. *R. solani* AG8 patches were associated with up to 30% grain yield loss in the US [17] and $59 m in annual losses to wheat in Australia [18]. AG2-1 is highly pathogenic on canola, causes severe hypocotyl rot on mustard and mild symptoms of hypocotyl rot on narrow-leafed lupin and clover, but failed to infect cereals, such as wheat, oats, barley, and ryegrass [16]. Other *R. solani* isolates cause severe diseases for other crops including potato [19], [20], canola [21], maize [22] and sugar beet [23], [24].

Current measures to control *R. solani* are non-specific, often not effective, and to date, breeding programs have struggled to identify plants with effective levels of resistance in the field. The broad host range of AG8, which causes bare-patch in wheat and lupin crops and is pathogenic in canola [15], [16], compounds the problem since crop rotations are often not able to help with disease control. A mutant wheat line with resistance to *R. solani* AG8 has been reported [25] although the mechanisms underlying this resistance remains to be elucidated. The heterologous expression of a germin-like protein from sugar beet in Arabidopsis also led to increased resistance against *R. solani* AG2 [26]. In addition, certain hypovirulent Rhizoctonia can protect plants against virulent Rhizoctonia isolates [27].

We have been interested in developing Arabidopsis as a model pathosystem to study plant responses to *R. solani* to take advantage of the extensive genetic and genomic resources. We have previously reported the transcriptional induction of a stress responsive promoter from an Arabidopsis glutathione S-transferse gene called *GSTF8* at an early stage of infection with *R. solani* [28]. The *GSTF8* promoter was induced early following challenge by *R. solani* AG8 (ZG1-1), a strain that is non-pathogenic on Arabidopsis. However, the GSTF8 induction was absent following inoculation with pathogenic strains, including AG2-1 (ZG5) [28]. These differences in susceptibility/resistance between AG8 and AG2-1 were seen consistently over different inoculum and infection conditions [28].

Here we report on our further investigations to understand the response of the plant to pathogenic and non-pathogenic strains of *R. solani* in Arabidopsis. Our focus has been on a) *R. solani* AG8 which has a wide host range and b) *R. solani* AG2-1, which has a narrower host range specialising on brassicas [29]. There is little variation in the response to either AG8 or AG2-1 among Arabidopsis ecotypes and resistance to AG8 is independent of common defence signalling pathways. Whole genome gene expression analysis at seven days post-infection identified genes that were differentially expressed in either the resistant or susceptible response including cell-wall associated genes, heat shock protein genes and other genes associated with oxidative stress responses. Functional data targeting key genes involved in reactive oxygen species (ROS) production demonstrated that specific NADPH oxidases are a critical component of resistance to *R. solani* AG8 in Arabidopsis, opening up new avenues to potentially control this destructive pathogen in various crop plants.

## Results

### Evaluation of the interaction of Arabidopsis ecotypes to *R. solani* AG8 and AG2-1

Col-0 is resistant to *R. solani* AG8 and susceptible to AG2-1 [28]. To determine if there is any variation in these responses in different Arabidopsis germplasm, 36 Arabidopsis ecotypes were screened against AG8 and AG2-1. The 36 ecotypes represented a diverse sample of the geographical and environmental range of this species [30]. Nine seedlings from each ecotype were treated with AG8, AG2-1 or non-infected control and survival was measured after two weeks. As shown in Table S1, all the ecotypes were resistant to AG8 with no observed symptoms. In contrast, all the ecotypes were susceptible to AG2-1, with survivorship in all cases being less than 33%.

### Resistance or susceptibility to *R. solani* AG8 or AG2-1 is independent of major defence signalling pathways

The large number of mutants that have been identified in specific signalling pathways in Arabidopsis have been helpful in elucidating key components of plant defence responses that operate in mediating resistance or susceptibility to specific pathogens (reviewed by [31], [32]). Mutants were compared that are affected in the signalling pathways for ethylene (ET) (*ein2*), jasmonic acid (JA) (*jar1*), salicylic acid (SA) (*NahG*, *cpr5*, *dnd1*, *npr1-5*), abscisic acid (ABA) (*aba1*, *abi1*) and auxin (*afb3*, *axr4-4*,*axr5-1*, *tir1-1*) as well as mutants defective in camalexin production (*pad3*, *pad4*). As shown in Table S2, all the mutants displayed similar levels of resistance to *R. solani* AG8 and susceptibility to AG2-1 compared to wild type. These results suggest that the common defence signalling pathways SA, ET, JA, auxin and ABA, or the phytoalexin camalexin, do not individually play a major role in resistance or susceptibility to *R. solani* in Arabidopsis.

### Gene expression changes in Arabidopsis in response to pathogenic and non-pathogenic *R. solani* isolates

**Figure 1:**
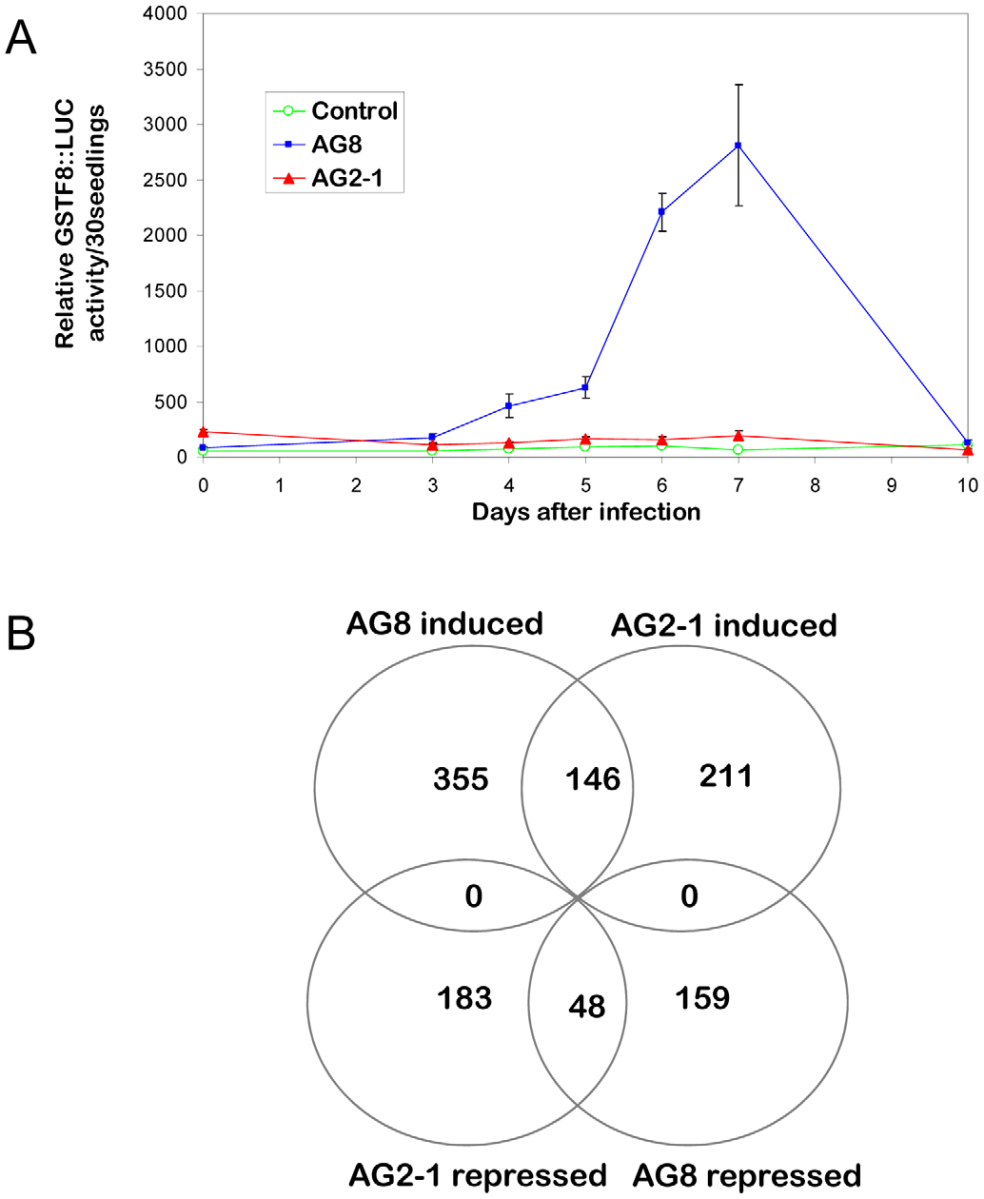
Microarray analysis of Arabidopsis genes differentially expressed following infection with *R. solani* AG8 or AG2-1. *A*. *In vivo* luciferase assay of Arabidopsis GSTF8::luciferase seedlings following infection with R. solani strain AG8, AG2-1 or non-infected control. The average bioluminescence values, measured for three plates containing 30 individual seedlings, are expressed in relative light units per seedling along with the standard errors. *B.* Venn diagram of the numbers of array elements on the Arabidopsis Affymetrix ATH1 Genome array differentially expressed at *P adjusted rate* ≤0.05 and induced or repressed by a factor of at least 2 by *R. solani* AG8 or AG2-1infection.

Given the novelty of the transcriptional regulation occurring in Arabidopsis following *R. solani* challenge, genome-wide expression analysis using Affymetrix ATH1 chips were performed to elucidate new pathways that may be mediating the high levels of resistance to *R. solani* AG8 and susceptibility to AG2-1. Gene expression patterns were compared in Arabidopsis plants inoculated with AG8, AG2-1 or non-infected control. As *R. solani* does not readily produce spores and primarily infects root tissue after mycelial growth through the plant growth matrix, the time of first contact between plant and fungus and the resulting plant response can be variable from experiment to experiment. Therefore, to determine the optimal time after inoculation for tissue collection, *GSTF8*::luciferase expression was used as a marker by monitoring luciferase expression in the transgenic *GSTF8*:: *luciferase* plants in parallel with wild type plants that had been simultaneously infected with AG2-1, AG8 or not infected. As shown in Figure 1A, high *GSTF8*::luciferase expression was seen at Day 7 after inoculation and so this time point was used for tissue collection.

Analysis of the expression data was focused on genes that were significantly differentially expressed, as determined by an adjusted *P* value of <0.05, and changed in expression level greater than two-fold. As shown in Figure 1B there were 501 genes that were up-regulated and 207 genes that were down-regulated in AG8 infected Arabidopsis seedlings compared to the non-infected treatment, using these criteria. For AG2-1 infected Arabidopsis seedlings there were 357 up-regulated genes and 231 down-regulated genes compared to the non-infected treatment. There were similarities in the response to the pathogenic and non-pathogenic strains with 146 and 48 genes up-regulated or down-regulated by both AG8 and AG2-1, respectively. Quantitative reverse transcriptase PCR (qRT-PCR) was used to check the validity of the microarray results for nine genes. The qRT-PCR and Affymetrix array results for these genes in general showed similar expression patterns although the RT-PCR assay revealed higher induction values than the Affymetrix array for some of the genes (Figure S1).

The Browser-based Functional Classification SuperViewer for Arabidopsis Genomics (http://bar.utoronto.ca/ntools/cgi-bin/ntools_classification_superviewer.cgi) was used to classify the genes that were differentially expressed following inoculation with AG2-1 or AG8. The results for each classification were calculated as follows: (Number_in Class input_set/Number_Classified input_set)/(Number_in_Class reference_set/Number Classified reference_set). It should be noted that with Superviewer, a given gene can belong to more than one group of classification. The results for AG2-1 vs non-infected control and AG8 vs non-infected control are presented in Figure S2 and S3. The classes of genes with the most number of members differentially expressed were quite similar for both the pathogenic and non-pathogenic *R. solani* strains. In the case of up-regulated genes the top three classes were genes that respond to biotic and abiotic stimuli, genes encoding extracellular proteins and stress response genes. In the case of down-regulated genes, the top three classes were cell wall related-genes, stress response and genes encoding extracellular proteins. However, when the specific genes that were differentially expressed were examined for these major gene classes we found many examples where distinct genes were being differentially expressed in response to the non-pathogenic versus the pathogenic *R. solani* strains.

**Figure 2:**
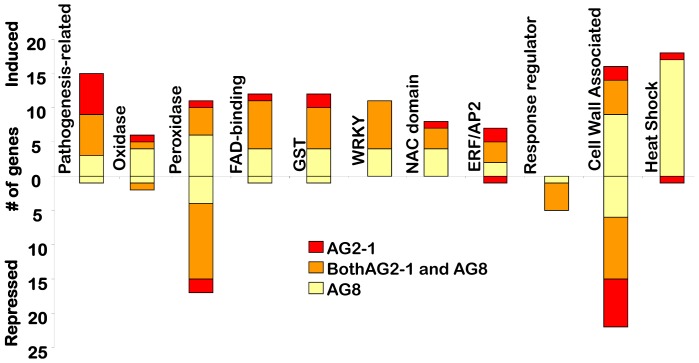
The number of genes affected by AG8 and AG2-1. The number of genes within selected families determine by containing the key words ‘oxidase’ ‘peroxidase’, ‘FAD-binding’, ‘GST’, ‘pathogenesis-related’, ‘ERF/AP2’, ‘NAC domain’, ‘response regulator’, ‘WRKY’, ‘heat shock/protein’ and cell wall identified on the Arabidopsis Affymetrix ATH1 Genome Array that were differentially expressed at P adjusted rate ≤0.05 and induced or repressed by a factor of at least two by *R. solani* AG8 or AG2-1infection. Of the 236 genes containing the key word ‘ubiquitin’, none were induced or repressed (data not shown). Genes were grouped into ‘cell wall’ criteria based on the program, mapman [50]. In some cases, two or more genes are identified from each array element name, and these are depicted as one gene.

To explore the differences and similarities between the responses to AG2-1 and AG8 further, the expression profiling results for AG2-1 infected tissue were directly compared with that obtained for AG8 infected tissue. The analysis revealed 222 genes were up-regulated and 146 were down-regulated in AG8 relative to AG2-1. Using this information, we focused on genes that could be grouped in the following classes; a) pathogenesis-related proteins b) genes linked to oxidative stress c) transcription factors, d) cell wall associated-proteins and e) heat shock proteins. In each case the degree of overlap between plant responses to the non-pathogenic AG8 and the pathogenic AG2-1, as well as specific differences were examined. The results of this analysis are presented in Figure 2 and Tables S3 and S4.

In Arabidopsis pathogenesis-related (PR) proteins fall into 17 groups [33], [34]. Both *R. solani* pathogens triggered the induction of pathogenesis-related genes when compared to the non-infected control; these included *PR1-like*, *PR4*, *PR5* and chitinase genes. *BGL2 (PR2)* was only induced by AG8 while *PR1* was only induced by AG2-1. When PR gene expression was directly compared between AG2-1 versus AG8 infected tissue, three PR genes were identified (*PR1-like* and two other putative PR genes) that were induced >two-fold by the pathogenic strain, AG2-1 compared to AG8 infection.

In the group of genes linked to oxidative stress, four gene families were targeted in the list of genes significantly differentially expressed; oxidases, peroxidases, flavin adenine dinucleotide (FAD)-binding proteins and Glutathione-S transferases (GSTs),

Oxidases that were induced specifically by AG8 included an alternative oxidase (*Aox1D*) and a respiratory burst oxidase homolog (*AtrbohD*) that is a key enzymatic subunit of the plant NADPH oxidase. The NADPH oxidases are involved in the generation of ROS which act as signals that mediate responses to infection and can also act directly on the invading pathogen [35], [36]. Interestingly, *AtrbohD*, has previously been linked to plant defence against fungal and bacterial pathogens [37].

Peroxidases play diverse roles in many aspects of plant biology including the generation of ROS and polymerisation of cell wall components [38]. A number of peroxidase family members were induced by either AG2-1 or AG8 compared to the non-infected control and this was more pronounced for AG8 (10 genes vs four). The peroxidase gene family also had a large number of members whose expression was repressed following both AG2-1 and AG8 inoculation when compared to the non-infected control.

Flavin adenine dinucleotide (FAD)–binding proteins have a role in electron transfer, are found in a number of oxidase complexes and may have a role in monitoring cellular redox changes [39], [40]. There were seven FAD-binding proteins genes induced by both AG2-1 and AG8 compared to the non-infected control. When AG8 and AG2-1 inoculated tissue were compared, four FAD-binding protein genes were induced >two fold by AG8, but only one FAD-binding protein gene was induced by AG2-1.

GST-family members have also been linked to oxidative stress [41], [42]. Four GST genes were induced by AG8 and two were induced by AG2-1 with a further six being induced by both AG8 and AG2-1. When GST expression was compared between AG2-1 and AG8 infected tissue, seven GSTs were induced by AG8 and five GSTs were induced by AG2-1.

The regulation of the temporal and spatial expression patterns of specific defence genes is a critical part of the plant defence response [43], [44]. Thirty nine (AG8) and 24 (AG2-1) transcription factors were induced >two fold compared to the non-infected control. The majority of these genes (19) were induced by both AG8 and AG2-1. Four families stood out in terms of the number of members that were differentially expressed 1) The WRKY family, which play roles in various aspects of plant biology including in plant defence (reviewed by [45]). 2) The NAC domain transcription factors which have been associated with acting as a master switch for secondary cell wall thickening [46]. 3) The AP2/ERF family of transcription factors which are strongly linked to pathogen responsive gene expression [47]. 4) The Arabidopsis response regulators that are involved in signal transduction for example, those mediated by cytokinins and ethylene [48], [49]. Response regulator transcription factors were the only family of transcription factors where the expression of a substantial number of members was down-regulated by both AG2-1 and AG8.

Genes encoding cell wall-associated proteins were one of the largest groups of genes responding to *R. solani* inoculation and were analysed using Mapman [50]. As shown in Figure 2 and Table S3, significant numbers of cell wall associated proteins were differentially expressed following inoculation with AG8 and/or AG2-1. The largest number of genes up regulated was seen with AG8 (eight genes), although there was also a substantial number of cell wall associated proteins induced by both AG8 and AG2-1 (five genes). In terms of gene repression, the largest number of changes was seen with cell wall associated genes that were repressed by both AG2-1 and AG8 (nine genes). A comparison of cell wall associated proteins that were differentially expressed between AG2-1 and AG8 infected tissue revealed that 16 genes were induced >two-fold in AG8 compared to AG2-1 but only four genes were induced >two-fold in AG2-1 compared to AG8. The AG8 induced genes included xyloglucan endotransglycosylases, pectinesterases and expansins (Tables S3 and S4).

Heat shock proteins (HSP) are molecular chaperones that are required for maintenance and restoration of protein homeostasis during stress conditions [51]. The major HSPs synthesized by eukaryotes belong to five conserved classes: HSP100, HSP90, HSP70, HSP60 and small (sm) HSP [51]. Interestingly, the expression of many HSP genes were induced by AG8 (17 genes), compared to the non-infected control, in many cases strongly as shown in Table 1. In contrast there were no HSP genes that were induced by AG2-1 compared to the non-infected control. SmHSP (∼16–30 kDa) family members were the class of HSPs that showed the most striking response to AG8, with five out of the seven HSPs that were induced greater than 10-fold by AG8 belonging to this class (Table 1). When AG8 and AG2-1 infected tissue were compared, 15 HSP genes were induced >two fold by AG8 but no HSP genes were expressed to a higher degree in AG2-1 infected tissue.

**Table 1:** List of Heat Shock Protein Genes Induced >two fold by AG8.

AG8	AG2-1	Heat shock Protein Gene
53.5	NS	AT3G46230 ATHSP17.4 (Arabidopsis thaliana heat shock protein 17.4)
52.3	NS	AT5G12030 AT-HSP17.6A (Arabidopsis thaliana heat shock protein 17.6A)
24.7	NS	AT1G53540 17.6 kDa class I small heat shock protein (HSP17.6C-CI) (AA 1-156)
20.8	NS	AT1G59860;AT1G07400 [AT1G59860, 17.6 kDa class I heat shock protein (HSP17.6A-CI)];[AT1G07400, 17.8 kDa class I heat shock protein (HSP17.8-CI)]
15.6	NS	AT5G52640 HSP81-1 (HEAT SHOCK PROTEIN 81-1); ATP binding/unfolded protein binding
15.4	NS	AT5G12020 HSP17.6II (17.6 KDA CLASS II HEAT SHOCK PROTEIN)
10.3	NS	AT1G74310 ATHSP101 (HEAT SHOCK PROTEIN 101); ATP binding/ATPase
10.0	NS	AT2G29500 17.6 kDa class I small heat shock protein (HSP17.6B-CI)
8.1	NS	AT1G52560 26.5 kDa class I small heat shock protein-like (HSP26.5-P)
8.0	NS	AT5G51440 23.5 kDa mitochondrial small heat shock protein (HSP23.5-M)
7.3	NS	AT3G12580 HSP70 (heat shock protein 70); ATP binding
4.3	NS	AT1G16030 HSP70B (heat shock protein 70B); ATP binding
3.8	NS	AT3G08970 DNAJ heat shock N-terminal domain-containing protein
3.5	NS	AT2G20560 DNAJ heat shock family protein
2.3	NS	AT5G02490 heat shock cognate 70 kDa protein 2 (HSC70-2) (HSP70-2)
2.3	NS	AT4G25200 ATHSP23.6-MITO (MITOCHONDRION-LOCALIZED SMALL HEAT SHOCK PROTEIN 23.6)
2.1	NS	AT3G09440 heat shock cognate 70 kDa protein 3 (HSC70-3) (HSP70-3)

Values list fold induction, if significant. NS: Not significantly induced greater than two-fold by AG2-1 (p<0.05).

### Functional analysis reveals a role for respiratory burst oxidase homologues in resistance to *R. solani*

**Figure 3:**
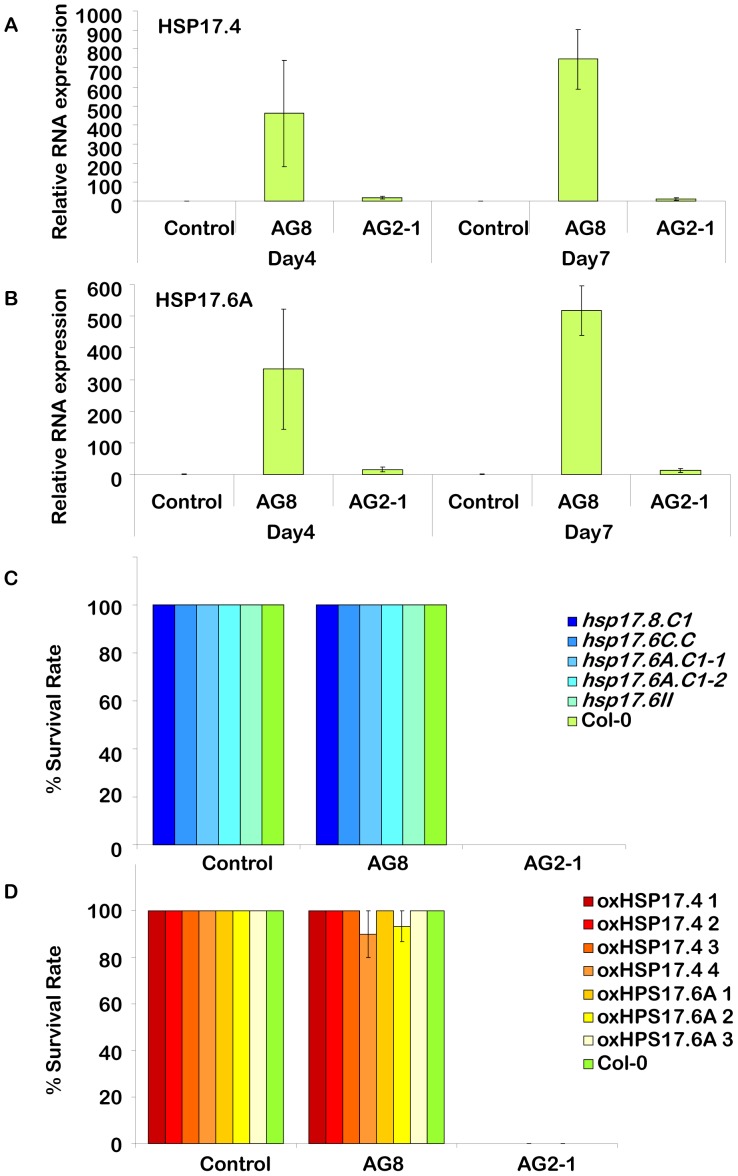
Analysis of the role of HSPs in the response to *R. solani*. Quantitative real time PCR of the genes *A. HSP17.4* and *B. HSP17.6A*. Three biological replicates and two technical repeats were analysed at each time point (four and seven day after infecting 1 week old seedling) and treatment (non-infected control, AG8 and AG2-1). The average of each time point and treatment is presented with standard error bars. Cyclophilin was used as the reference gene to normalize expression. Day 4 (control) is standardized to equal one and all other treatments and timepoints reflect their fold induction compared to this point. Survival rate of *C.* loss of function and *D.* gain of function. Arabidopsis lines. Col-0 and the homozygous SALK lines, *hsp17.8.C1* (SALK_059920C– At1g07400), *hsp17.6C.C1* (SALK_056782C – AT1g53540), *hsp17.6A.C1-1* (SALK_152961C – At59860), *hsp17.6A.C1-2* (SALK_056925C – At59860) and *hsp17.6II* (SALK_043928C - At5g12020) were tested for survivorship against non-infected control, *R. solani* AG8 and AG2-1. Four independent lines overexpressing *HSP17.4* (oxHSP17.4 lines 1–4), and three independent lines overexpressing *HSP17.6* (oxHSP17.6 lines 1–3) were tested for survivorship against non-infected control, *R. solani* AG8 and AG2-1.

To further explore the roles of some of the differentially expressed genes identified from the gene expression study that may have a role in ROS, follow-up RT-PCR and reverse genetic experiments were performed focusing on HSP genes and oxidases. Quantitative RT- PCR experiments were used to analyse the response of HSP17.4 and HSP17.6A, which had the highest fold induction among HSP genes following AG8 infestation. As shown in Figure 3A and 3B, the expression of these genes was already induced in AG8 infected tissue four days post inoculation by several hundred fold and both HSP genes were still highly induced by day seven, the time point used for the Affymetrix studies. In striking contrast, inoculation with AG2-1 only caused a relatively small induction at either time point. To analyse the role HSPs may play in resistance to *R. solani* we analysed both loss-of-function and gain-of-function mutants in specific HSP genes. For the loss-of-function mutants we analysed five individual SALK T-DNA insertion lines in four of the HSP genes but found the response of these loss-of-function HSP mutants to either *R. solani* AG8 or AG2-1 was similar to wild type (Figure 3C). Gain-of-function mutants for HSP 17.4 and 17.6A were generated through overexpression in transgenic Arabidopsis using the strong CaMV 35S promoter and high levels of overexpression were achieved. These HSP overexpression lines were not affected in their resistance or susceptibility to *R. solani* AG8 or AG2-1, respectively (Figure 3D).

**Figure 4:**
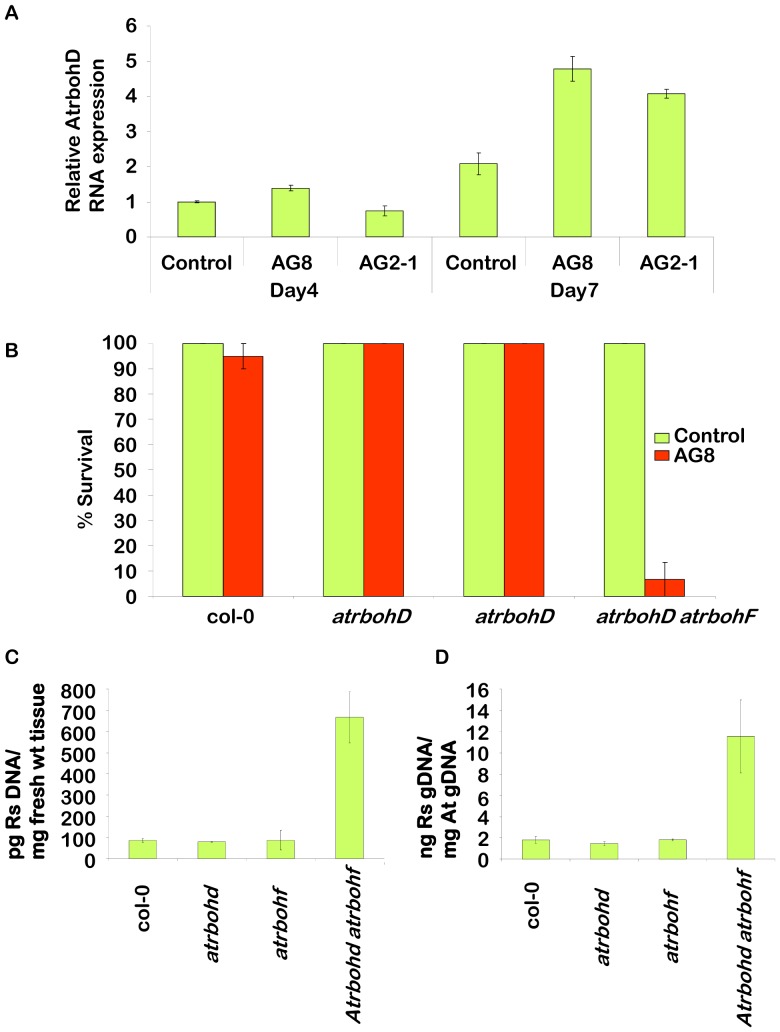
Analysis of the role of NADPH oxidases in resistance to *R. solani* AG8. *A.* Real time PCR of the genes, *AtrbohD* and *AtrbohF*. Three biological replicates and two technical repeats were analysed at each time point (four and seven day after infecting one week old seedlings) and treatment (non-infected control, AG8 and AG2-1). The average of each time point and treatment is presented with standard error bars. Cyclophilin was used as the reference gene. Day four (control) is standardized to equal one and all other treatments and time points reflect their fold induction compared to this point. A star above the bar is indicated if the value is significantly different from the control (Dunnett's test p<0.05). *B.* Survivorship of homozygous *atrbohD* (SALK_005853C – At5g47910), homozygous *atrbohF* (SALK_034674C – At1g64060), the double knockout *atrbohD atrbohF* (CS9558 - At5g47910 & At1g64060) and Col-0 in response to *R. solani* AG8 compared to WT. Seedling survivorship was recorded two weeks after planting seedlings into pre-infected pots. *C.* Relative *R. solani* genomic DNA in homozygous *atrbohD*, homozygous *atrbohF*, the double knockout *atrbohD atrbohD* and Col-0 in response to *R. solani* AG8 compared to WT at nine days after infection. Quantification of *R. solani* is presented as ng fungal DNA/µg plant DNA and *D.* ng fungal DNA per mg of infected tissue used for DNA extraction.

The other class of genes examined in more detail were respiratory burst oxidase homologues. We focused initially on *AtrbohD* which the Affymetrix analysis had shown was significantly induced 2.3 fold by AG8 infection. Further expression analysis using quantitative qRT-PCR (Figure 4A) confirmed that the gene was induced by AG8 at day 7 after infection and revealed a small induction at day 4. The Affymetrix data showed that *AtrbohD* was induced 1.4 fold by AG2-1 which was below our cut-off of 2-fold inductions and that the expression of *AtrbohF* was not significantly affected. However, the more sensitive qRT-PCR confirmed that this gene was induced by AG2-1 at day 7 but not at day 4. A loss-of-function mutant for *AtrbohD* involving a T-DNA insertion (*atrbohD*) displayed the same levels of resistance to *R. solani* AG8 as wild type (Figure 4B). *AtrbohD* has been shown to function together with another member of the family, *AtrbohF* in the accumulation of ROS following pathogen attack [37]. A loss-of-function mutant for *AtrbohF* (*atrbohF*) also had no effect on resistance to AG8 (Figure 4B). However, the double mutant *atrbohD atrobohF* displayed a high degree of susceptibility to AG8 with just 7% survival at two weeks (Figure 4B). Measurement of the ratio of AG8 genomic DNA to plant fresh weight (Figure 4C) or Arabidopsis genomic DNA (Figure 4D) in wild type and the mutant plants at nine days following AG8 infection (prior to the dramatic affects on seedling death), demonstrated that *R. solani* is five – eight times more abundant in the double mutant *atrbohD atrobohF* than wild type or single mutant plants. The observation of increased *R. solani* growth coupled with plant death in the *atrbohD atrobohF* double mutant following AG8 infection, demonstrates that these genes play an important role in resistance to AG8 in Arabidopsis.

## Discussion

While Rhizoctonia species are important pathogens for many different crops, the diversity within Rhizoctonia species, the wide host range of some isolates and the lack of useful genetic resistance has meant that plant breeders have had limited success in identifying plants with effective resistance to these soil born necrotrophic fungal pathogens and incorporating these into breeding programs. This problem is compounded by the fact that fungicide use is not economical nor particularly effective against many soil-borne pathogens [8]. Alternative approaches are needed to understand how plants respond to *R. solani*. The development of a pathosystem involving Arabidopsis, the premier plant model system, has the potential to provide valuable information on plant responses to pathogenic (AG2-1) and non-pathogenic (AG8) strains of *R. solani*. One of the findings of this study was that Arabidopsis germplasm, which has been shown to have considerable variation in response to the defence signal SA [52], had little variation in terms of susceptibility to AG2-1 or resistance to AG8.

The response of Arabidopsis to *R. solani* strains AG2-1 and AG8 was not affected by mutants in a number of major defence signalling pathways in contrast with what has been observed with some other necrotrophic fungi in Arabidopsis. For example, resistance to *F. oxysporum* and *B. cinerea* requires the ET, JA, and SA signalling pathways [53] but mutants in these pathways had no effect on susceptibility/resistance to either AG2-1 or AG8. The ET findings were surprising as ET insensitive mutants in soybean (*etr1*, *etr2*) and *Medicago truncatula (sickle)* resulted in enhanced susceptibility to *R. solani* [54], [55], [56]. However, our results are consistent with previous results using knock out lines for an ET inducible Arabidopsis transcription factor, AtERF14, which resulted in higher susceptibility to *F. oxysporum* but had no effect on AG8 inoculation [57]. Others have observed that the effect of ET on plant responses to fungal pathogens can vary in different plant species [58].

There has been only limited work using transcriptomic approaches to study plant responses to *R. solani*, with the only genomic study being performed on a moderately resistant rice cultivar, infected with *R. solani* AG1 [59]. Given the association of the *GSTF8* promoter with the strong resistance to *R. solani* AG8, we used the *GSTF8::luciferase* reporter system to monitor plant responses in real time after inoculation, thereby allowing us to harvest physiologically synchronised tissue for the large scale expression profiling experiments. Although only one time point (7days) was investigated, some of the key findings from the expression profiling experiments were that while AG8 is non-pathogenic on Arabidopsis it was still able to cause large changes in host gene expression. This indicates the plant was able to recognise AG8 as a potential pathogen and mount a response that correlated with effective resistance.

One of the pronounced differences between AG2-1 and AG8 infested tissue was the expression of genes encoding cell wall-related proteins. We were interested in proteins known to be involved in cell wall modification such as xyloglucan endotransglycosylases, expansins, polygalacturonases and pectin methylesterases, particularly in light of possible cell wall modifications that may be occurring through ROS mediated cross-linking. These were well represented among the cell wall proteins that were differentially expressed between AG8 and AG2-1 infected plants. Modifications of pectic polysaccharides through mutations or expression of polygalacturonases and PME inhibitor proteins *in planta* have been shown to affect the outcome of various plant pathogen interactions [60], [61], [62]. The results are also consistent with observations that correlate cuticle & cell wall thickness with quantitative increases in resistance to *R. solani* [63], [64].

The gene family with the largest transcriptional difference between AG2-1 and AG8 infected tissue at seven days post-infection was the HSPs which were up-regulated exclusively in the non-pathogenic interaction, many by 10-fold or more. This was most notable for a specific class of HSPs, the small HSP class which are the most complex class of HSP in plants and may play a role in survival and recovery from abiotic stress [65]. Small HSP have been linked to plant defence including the observation of enhanced susceptibility to *Ralstonia solanacerum* in tobacco in a HSP17 silenced line [66]. There is also evidence linking other HSPs to plant defence such as the HSP90 family (heat shock protein 90 kD) which has a role in NB-LRR type R protein triggered immunity against *Pseudomonas syringae* [67]. However, when we analysed individual T-DNA insertion liness in four of the HSPs to determine if an alteration in their individual expression affected the plant's response to *R. solani*, in each case their response to *R. solani* AG8 or AG2-1 was similar to wild type. However it remains to be determined if double or multiple HSP mutants would affect *R. solani* resistance. In addition, overexpression of HSP17.4 and 17.6, the two HSPs with the largest induction following AG8 infection, did not alter the plant's response to *R. solani*. Collectively, this suggests that the individual HSPs do not have a direct role in the plant's response to *R. solani* but given the large number of HSPs induced specifically by AG8, HSPs may be part of the effect rather than the cause of the plant's resistance to *R. solani*.

One of the largest functional groups of genes responding to *R. solani* challenge was those associated with oxidative stress including oxidases, FAD binding, peroxidases and GST gene families. One of the main sources of ROS during pathogen infection is from the *Rboh*-NADPH oxidases located in the plant plasma membranes. The NADPH oxidase mutants *atrbohD* and *atrbohF* can both significantly reduce ROS production and affect plant disease resistance; however, the effects of these *rhobh*-NADPH oxidases on plant resistance have been variable and are dependent on the specific plant-pathogen interaction [36], [68]. For example, the double mutant, *atrbohD atrobhF* displayed reduced cell death after infiltration with avirulent *P. syringae* without any effect on the growth of the bacterium [37]. Meanwhile, the same double mutant exhibited enhanced resistance to a virulent strain of the oomycete *Hyaloperonospora arabidopsidis* [37]. In contrast, the silencing of two *rhobh*-NADPH oxidase homologs in *Nicotiana benthamiana (NbrbohA and NbrbohB)* showed enhanced susceptibility to the oomycete pathogen *Phytophthora infestans* [69].

The conflicting reports of the role of NADPH oxidases in pathogen defence may stem from the dual role of the AtRBOH-derived ROS in both triggering and suppressing cell death. Although AtRBOH-derived ROS has been shown to potentiate cell death at the site of infection, these ROS can also prevent the uncontrolled spread of cell death from the infection site by regulating JA/ET and SA levels in neighbouring cells [35], [70]. The role of RBOHD proteins in the defence against necrotrophs has been analysed in an Arabidopsis-*A. brassicicola* pathosystem [70]. The *atrbohD* mutant exhibited a spreading necrosis phenotype that was reminiscent of enhanced plant susceptibility to this pathogen. However, a measure of fungal biomass showed that fungal colonization of the mutant was similar to wildtype, suggesting that AtRBOHD was a regulator of cell death rather than playing a role as a fungal resistance factor in this interaction [70]. We report transcriptional regulation of *AtrbohD*, in response to *R. solani* AG8, but other factors also play a role in regulation of NADPH oxidases such as phosphorylation [71] and S-nitrosylation [72]. In this paper we show that individual mutants in *atrbohD* and *atrbohF* had no disease phenotype following *R. solani* infection, but the *atrbohD atrbohF* double mutant was substantially more susceptible to *R. solani* AG8 (Figure 4). In this case, the enhanced susceptibility to AG8 was accompanied by increased fungal biomass in the double mutant. This indicates that in the Arabidopsis-*R. solani* AG8 pathosystem, AtROBHD and AtROBHF are essential players in plant resistance to this necrotrophic root pathogen. Important questions remain as to how Arabidopsis is able to recognise and respond effectively to AG8 while crop plants, including wheat, are not, and how AG2-1 can avoid/suppress the NADPH oxidase mediated defence. The Arabidopsis-*R. solani* pathosystem described herein provides an excellent opportunity to apply powerful genomic and genetic tools to further address these questions.

## Methods

### Plant Material

Ecotypes, SALK lines [73] and signal mutants were obtained from the ARBC: http://www.biosci.ohio-state.edu/~plantbio/Facilities/abrc/abrchome.htm. The transgenic line *GSTF8*:: *luciferase* contains the −783 *GSTF8* promoter fused to the *luciferase* reporter gene in the Columbia ecotype background as previously described [74]. To generate HSP17.4, HSP17.6A, AtEXLA1 and XTR7 overexpressing lines, primers (see Table S5) were used to generate a PCR product that was inserted into the vector, pDONRzeo (Invitrogen: www.invitrogen.com) by gateway cloning. The inserts were transferred into the vectors, pCH184 (oxHSP17.4 lines 1 and 2 and oxHSP17.6A lines 1–3, AtEXLA1 and XTR7 lines) or pDEST-NOSBar-35s (oxHSP17.4 lines 3 and 4) and transformed in the *Agrobacterium tumefaciens* strain GV3101 before transforming into Arabidopsis using the standard floral dipping technique [75]. Seeds were selected using kanamycin (with pCH184 vector) or BASTA (with pDEST-NOSBar-35S vector). T3 seeds were used to test for responses to *R. solani*. Quantitative reverse transcription PCR (RT-PCR) was performed as previously described [76] and confirmed the overexpression of HSP17.4 and HSP17.6A in the transgenic lines (data not shown).

### *R. solani* and inoculation of plants

The source of *R. solani* strains, AG8 (ZG1-1; WAC10335) and AG2-1: (ZG5: WAC9767), its maintenance, production of *R. solani* infected millet seeds and bioluminescence techniques have been described previously [28]. Fine vermiculite or soil was infected by placing four *R. solani*-coated millet seeds on the surface of each pot. The *R. solani* strains were left to grow for seven days before planting. Five to nine seedlings were grown in each pot and were replicated at least 3 times. One week old Arabidopsis seedlings were transferred from sterile MS-agar medium to pre-colonised pots, and grown in a growth cabinet (22°C, 16 hr light∶ 8 hr dark photoperiod). Survivorship rates were then scored after 14 days inoculation. Plants were defined as R (resistant) if no lethality occurred (survivorship of 100%) and S (susceptible) if survivorship was less than 33%. There were no plants observed in the moderate-susceptible range (survivorship 34–99%).

### Affymetrix analysis

One-week-old Col-0 seedlings grown on vertical MS plates were treated by touching the root/crown region by a toothpick containing mycelia from *R. solani* grown on PDA [28]. At the same time, *GSTF8*::*luciferase* transgenic plants were grown on MS plates with luciferin and luciferase activity was measured at day 0. 3, 4, 5, 6, 7 and 10 days. Based on a measurement of maximum *GSTF8*::luciferase activity, whole seedlings were collected seven days after treatment. EAch treatment involved 100 mg tissue (∼3 seedlings) from the non-infected control, AG8 and AG2-1 treated plants. RNA was extracted using the Qiagen RNA easy kit following manufacturer's protocol (www.qiagen.com). Whole plant RNA (15 ug for each sample) was sent to the Australian Genome Research Facility Ltd. (www.agrf.org.au) for labelling and hybridization to Affymetrix Arabidopsis ATH1 genome arrays. Three biological replicates were used for each treatment. Probability values were adjusted by the Benjamini-Hochberg method [77]. Resulting signal data was analysed using the Limma Bioconductor package in R (http://www.bioconductor.org) [78]. The array data was normalized using the EXPRESSO function.

A robust multi-chip average was calculated using quantile normalization, background correction and the median polish method as recommended by [79] and [80]. Gene expression was referred to significant, if the adjusted P value was <0.05 and the expression greater than two fold. The function of the genes was determined by submission of the array element names to the TAIR website: http://www.arabidopsis.org/tools/bulk/microarray/index.jsp. Gene families were grouped by containing key words. Genes were grouped into ‘cell wall’ criteria based on the program, mapman [50].

Microarray data generated from this article is Miame Compliant [81] and the data discussed in this publication have been deposited in NCBI's Gene Expression Omnibus [82] and are accessible through GEO Series accession number GSE26206 (http://www.ncbi.nlm.nih.gov/geo/query/acc.cgi?acc=GSE26206).

### Real-time PCR

RNA extraction, cDNA synthesis and quantitative real-time PCR (qRT-PCR) to measure RNA expression was performed on the Biorad MyIQ as previously described [76] with annealing temperatures of 60°C. The primers are listed in Table S5. Each primer pair amplifies a specific gene as determine by BLAST sequence analysis.

Quantification of *R. solani* genomic DNA was determined using quantitative real time PCR with primers annealing to the internal transcribed region of *R. solani* AG8 rDNA [83] using the Biorad MyIQ an annealing temperatures of 60°C. Sequences of primers are listed in Table S5. One week old seedlings were planted in *R. solani* AG8 infected soil, and DNA was extracted nine days after infection [84]. The quantitative PCR utilised the primers generated against AG8 5.8s rDNA (Table S5) and the method was similar as that for measuring cDNA except 100 ng of genomic DNA was added instead of cDNA. The quantity of *R. solani* gDNA was standardized against mg fresh weight tissue and the quantity of Arabidiopsis β-tubulin genomic DNA.

## Supporting Information

Figure S1 **RNA fold induction as determined by Affymetrix analysis and quantitative RT-PCR.** The RNA fold inductions of seedlings infected with AG8 or AG8-1 compared with mock were determined for the genes, *GSTF7*, *PR1*, *WRKY33*, *PR4*, *Bos1*, *ChitB*, *Lox2*, *PDF1.2* and *PAD3*.
(TIF)

Figure S2 **Classes of genes induced by A) AG8 vs mock and B) AG2-1 vs mock.** Gene numbers have been categorized using The Browser-based Functional Classification SuperViewer for Arabidopsis Genomics. Values higher than 1 demonstrate that the number of genes in a specific classification group are more represented than random.
(TIF)

Figure S3 **Classes of genes repressed by A) AG8 vs mock and B) AG2-1 vs mock.** Gene numbers have been categorized using The Browser-based Functional Classification SuperViewer for Arabidopsis Genomics. Values higher than 1 demonstrate that the number of genes in a specific classification group are differentially expressed at a higher rate than uninduced.
(TIF)

Table S1 **Response of Arabidopsis ecotypes with R. solani AG8 and AG2-1.** Plants were either scored resistant (R, 100% survival), or susceptible (S, <33% survival).
(DOCX)

Table S2 **Response of Arabidopsis mutants to R. solani AG8 and AG2-1.** Plants were either scored resistant (R, 100% survival), or susceptible (S, <33% survival).
(DOCX)

Table S3 **Affymetrix results of selected Arabidopsis genes of seedlings infected with** ***R.solani*** **AG8 vs AG2-1.** Genes with higher expression in *R. solani* AG8 infected tissue compared to *R. solani* AG2-1 infected tissue (adjusted P value<0.05 and a fold change >2.0).
(DOCX)

Table S4 **Affymetrix results of selected Arabidopsis genes of seedlings infected with** ***R.solani*** **AG2-1 vs AG8.** Genes with higher expression in *R. solani* AG2-1 infected tissue compared to *R. solani* AG8infected tissue (adjusted P value<0.05 and a fold change >2.0).
(DOCX)

Table S5 **List of relevant oligonucleotides.** Primer Sequences (5′ – 3′) used in quantitative RT-PCR and cloning.
(DOCX)
