# Supplementary figures and images for: Genetic and Genomic Analysis of Rhizoctonia solani Interactions with Arabidopsis; Evidence of Resistance Mediated through NADPH Oxidases
Source: PLoS One. 2013 Feb 25;8(2):e56814. doi: 10.1371/journal.pone.0056814 (PMC3581538; doi:10.1371/journal.pone.0056814)

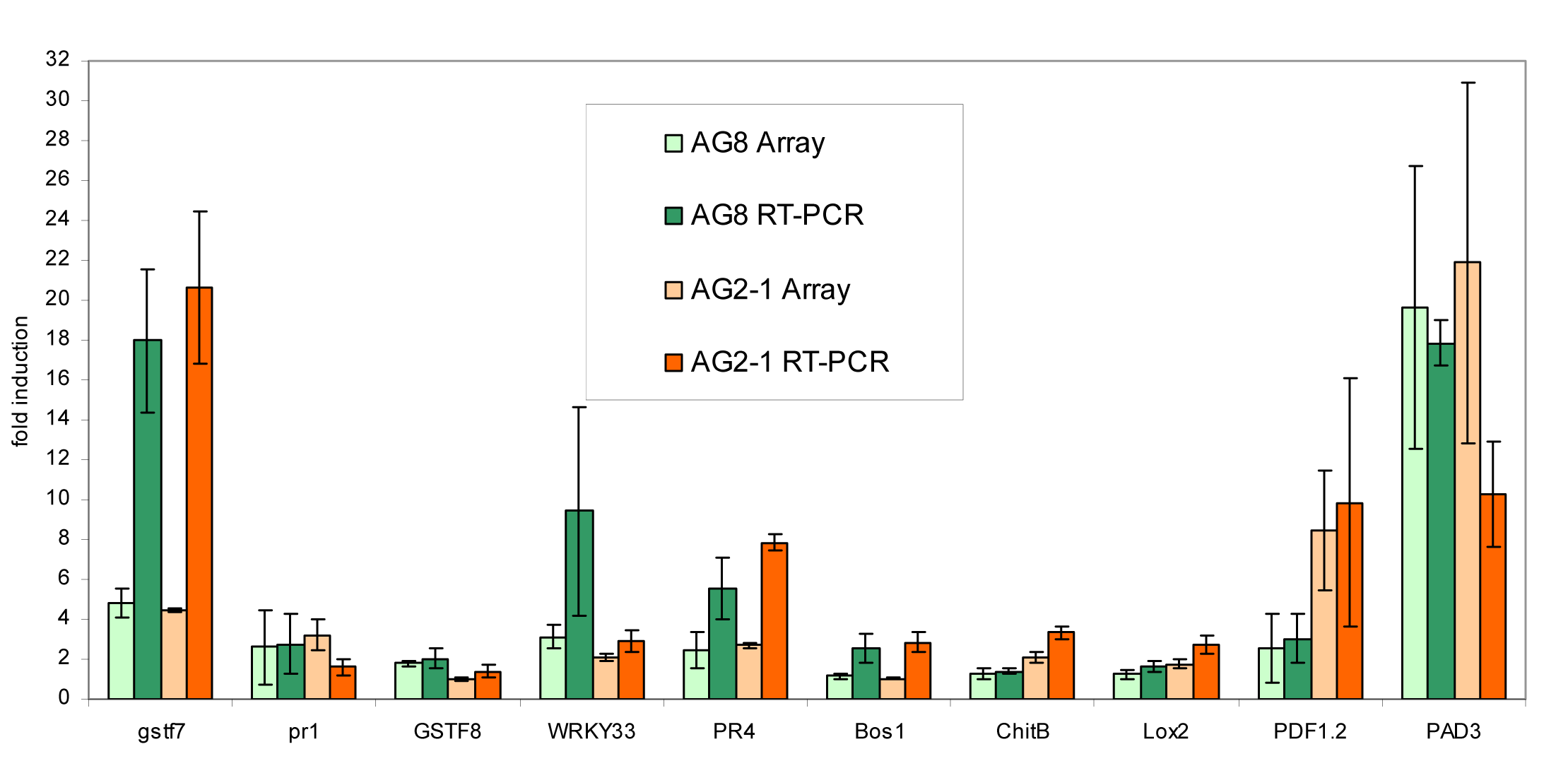

Supplement: Figure S1 — RNA fold induction as determined by Affymetrix analysis and quantitative RT-PCR. The RNA fold inductions of seedlings infected with AG8 or AG8-1 compared with mock were determined for the genes, GSTF7, PR1, WRKY33, PR4, Bos1, ChitB, Lox2, PDF1.2 and PAD3. (TIF) [file pone.0056814.s001.tif]

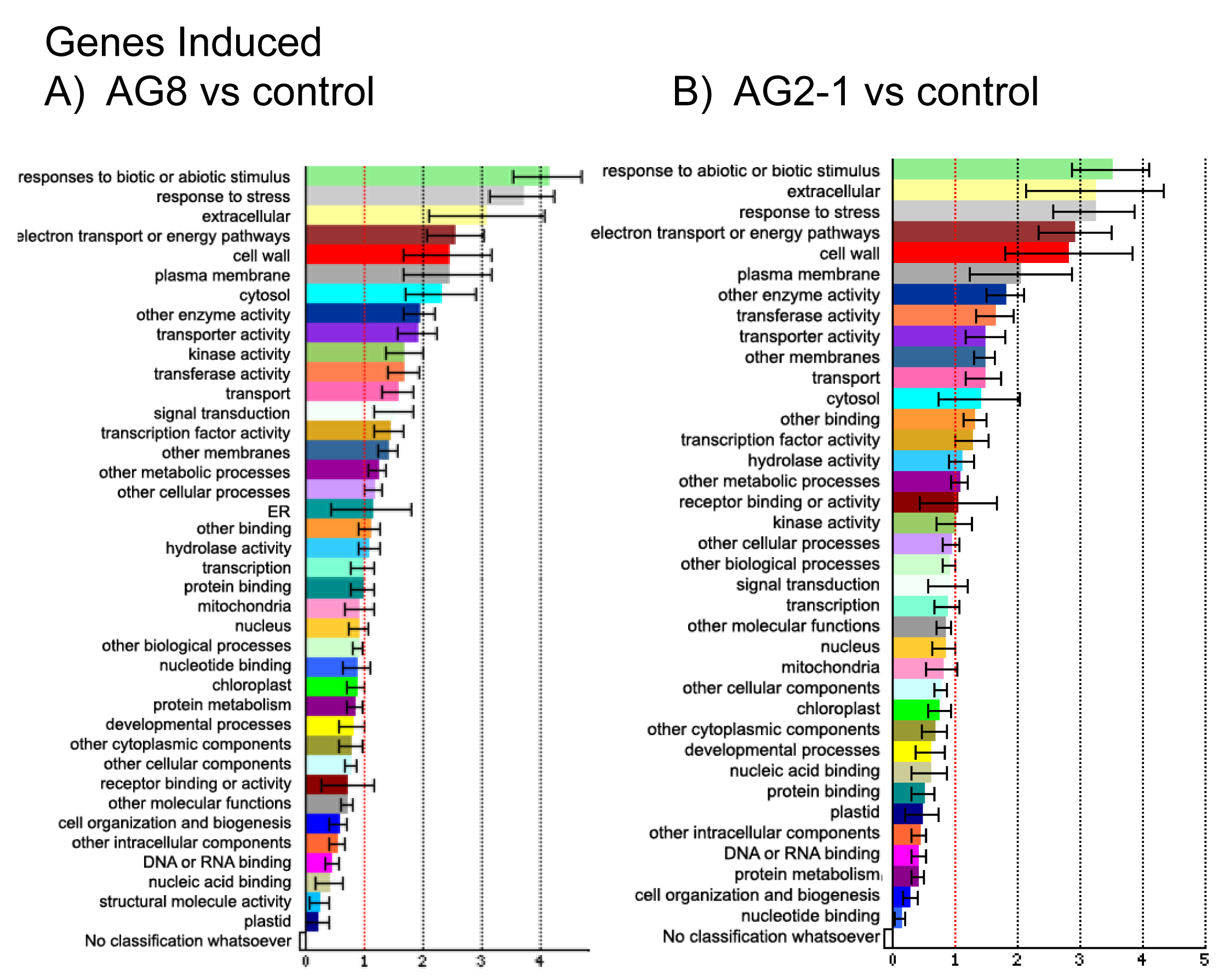

Supplement: Figure S2 — Classes of genes induced by A) AG8 vs mock and B) AG2-1 vs mock. Gene numbers have been categorized using The Browser-based Functional Classification SuperViewer for Arabidopsis Genomics. Values higher than 1 demonstrate that the number of genes in a specific classification group are more represented than random. (TIF) [file pone.0056814.s002.tif]

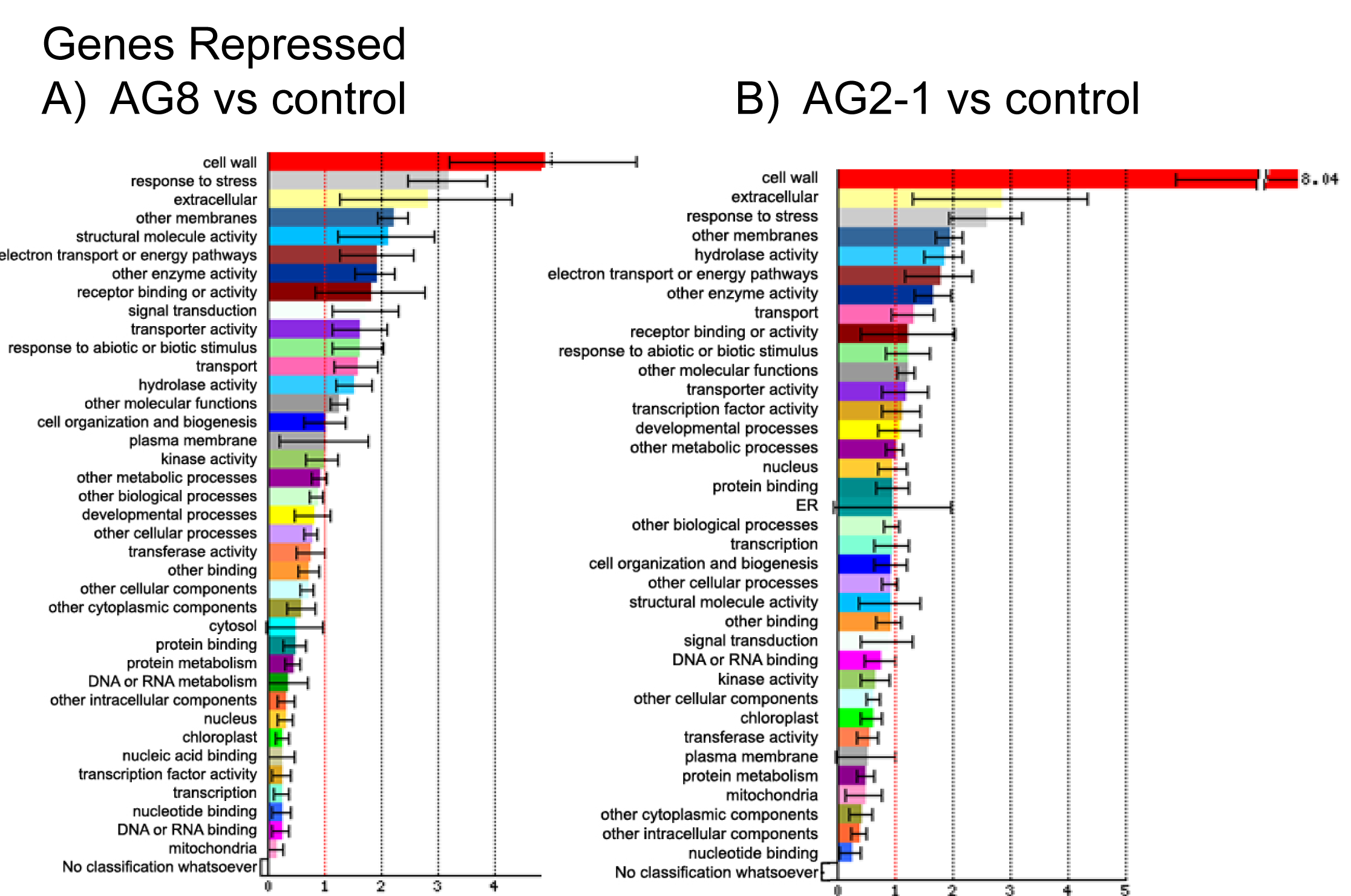

Supplement: Figure S3 — Classes of genes repressed by A) AG8 vs mock and B) AG2-1 vs mock. Gene numbers have been categorized using The Browser-based Functional Classification SuperViewer for Arabidopsis Genomics. Values higher than 1 demonstrate that the number of genes in a specific classification group are differentially expressed at a higher rate than uninduced. (TIF) [file pone.0056814.s003.tif]
