# Supplementary material for: Genetic and Genomic Analysis of Rhizoctonia solani Interactions with Arabidopsis; Evidence of Resistance Mediated through NADPH Oxidases
Source: PLoS One. 2013 Feb 25;8(2):e56814. doi: 10.1371/journal.pone.0056814 (PMC3581538; doi:10.1371/journal.pone.0056814)
Supplement: Table S1 — Response of Arabidopsis ecotypes with R. solani AG8 and AG2-1. Plants were either scored resistant (R, 100% survival), or susceptible (S, <33% survival). (DOCX) [file pone.0056814.s004.docx]

**Table S1**

S

R

Yo-0

S

R

Ws

S

R

Wi-0

S

R

Tac

S

R

Su-0

S

R

Sorbo

S

R

Sei-0

S

R

Rsch-0

S

R

Pog-0

S

R

Petergof

S

R

Oy-0

S

R

No-0

S

R

Mt-0

S

R

Mrk-0

S

R

Mr-0

S

R

Ma-0

S

R

Lip-0

S

R

Ler-0

S

R

Kondara

S

R

Kil-0

S

R

Kas-1

S

R

Ka-0

S

R

Hodja

S

R

Emal-1

S

R

Ei-2

S

R

Di-G

S

R

Di-1

S

R

Cvi

S

R

Col-5(gl1)

S

R

Cn-1

S

R

Can-0

S

R

Cal-0

S

R

Bs-1

S

R

Bla-10

S

R

Bl-1

S

R

An-1

S

R

Aa-0

S

R

Col-0

**AG2-1**

**AG8**

**Ecotypes**
