# Supplementary material for: Genetic and Genomic Analysis of Rhizoctonia solani Interactions with Arabidopsis; Evidence of Resistance Mediated through NADPH Oxidases
Source: PLoS One. 2013 Feb 25;8(2):e56814. doi: 10.1371/journal.pone.0056814 (PMC3581538; doi:10.1371/journal.pone.0056814)
Supplement: Table S2 — Response of Arabidopsis mutants to R. solani AG8 and AG2-1. Plants were either scored resistant (R, 100% survival), or susceptible (S, <33% survival). (DOCX) [file pone.0056814.s005.docx]

**Table S2**

| **Signal Mutants** | **AG8** | **AG2-1** |
| --- | --- | --- |
| aba1 | R | S |
| *abi1* | R | S |
| *afb3* | R | S |
| *axr4-1* | R | S |
| *axr5-1* | R | S |
| *dnd1* | R | S |
| *ein2* | R | S |
| *jar1* | R | S |
| *mpk4* | R | S |
| *NahG* | R | S |
| *npr1-5* | R | S |
| *pad3* | R | S |
| *pad4* | R | S |
| *tir1-1* | R | S |
