# Supplementary material for: Genetic and Genomic Analysis of Rhizoctonia solani Interactions with Arabidopsis; Evidence of Resistance Mediated through NADPH Oxidases
Source: PLoS One. 2013 Feb 25;8(2):e56814. doi: 10.1371/journal.pone.0056814 (PMC3581538; doi:10.1371/journal.pone.0056814)
Supplement: Table S3 — Affymetrix results of selected Arabidopsis genes of seedlings infected with R.solani AG8 vs AG2-1. Genes with higher expression in R. solani AG8 infected tissue compared to R. solani AG2-1 infected tissue (adjusted P value<0.05 and a fold change >2.0). (DOCX) [file pone.0056814.s006.docx]

**Table S3**

| AGI number | Probe set | fold | Gene name |
| --- | --- | --- | --- |
| ***Cytochrome*** | |  |  |
| AT5G58860 | 247765_at | 2.25 | CYP86A1 (Cytochrome P450, family 86, subfamily A, Polypeptide 1); oxygen binding |
| AT4G37370 | 253046_at | 2.02 | CYP81D8 (Cytochrome P450, family 81, subfamily D, Polypeptide 8); oxygen binding |
| ***GST*** |  |  |  |
| AT1G17180 | 262517_at | 3.20 | ATGSTU25 (Arabidopsis thaliana Glutathione S-transferase (class tau) 25); glutathione transferase |
| AT1G17170 | 262518_at | 2.27 | ATGSTU24 (ARABIDOPSIS THALIANA GLUTATHIONE S-TRANSFERASE (CLASS TAU) 24); glutathione transferase |
| ***Peroxidase*** |  |  |  |
| AT4G36430 | 246228_at | 3.04 | peroxidase, putative |
| AT1G68850 | 260035_at | 2.74 | peroxidase, putative |
| AT5G06730 | 250702_at | 2.73 | peroxidase, putative |
| AT5G05340 | 250798_at | 2.50 | peroxidase, putative |
| AT5G66390 | 247091_at | 2.47 | peroxidase 72 (PER72) (P72) (PRXR8) |
| AT4G33420 | 253332_at | 2.45 | peroxidase, putative |
| multiple | 263063_s_at | 2.10 | [AT2G18150, peroxidase, putative];[AT2G18140, peroxidase, putative] |
| ***FAD-binding*** | |  |  |
| AT5G44440 | 249053_at | 3.43 | FAD-binding domain-containing protein |
| AT1G30700 | 263228_at | 2.76 | FAD-binding domain-containing protein |
| AT5G44400 | 249046_at | 2.68 | FAD-binding domain-containing protein |
| AT1G30760 | 264527_at | 2.30 | FAD-binding domain-containing protein |
| ***Chitinase*** |  |  |  |
| none |  |  |  |
| ***Pathogenesis*** |  |  |  |
| none |  |  |  |
| ***ERF/AP2*** |  |  |  |
| AT1G21910 | 260856_at | 3.76 | AP2 domain-containing transcription factor family protein |
| ***NAC domain*** | |  |  |
| AT5G64530 | 247264_at | 3.54 | ANAC104/XND1 (Arabidopsis NAC domain containing protein 104); transcription factor |
| AT1G02220 | 264148_at | 2.06 | ANAC003 (Arabidopsis NAC domain containing protein 3); transcription factor |
| AT5G39610 | 249467_at | 2.04 | ANAC092/ATNAC2/ATNAC6 (Arabidopsis NAC domain containing protein 92); protein heterodimerization/ protein homodimerization/ transcription factor |
| ***Response Regulator*** | |  |  |
| none |  |  |  |
| ***WRKY*** |  |  |  |
| AT4G31800 | 253485_at | 2.42 | WRKY18 (WRKY DNA-binding protein 18); transcription factor |
| AT2G30250 | 267246_at | 2.08 | WRKY25 (WRKY DNA-binding protein 25); transcription factor |
| AT4G01250 | 255568_at | 2.05 | WRKY22 (WRKY DNA-binding protein 22); transcription factor |
| ***Heat Shock*** |  |  |  |
| AT5G12030 | 250351_at | 33.54 | AT-HSP17.6A (Arabidopsis thaliana heat shock protein 17.6A) |
| AT3G46230 | 252515_at | 30.13 | ATHSP17.4 (Arabidopsis thaliana heat shock protein 17.4) |
| AT1G59860;AT1G07400 | 262911_s_at | 16.71 | [AT1G59860, 17.6 kDa class I heat shock protein (HSP17.6A-CI)];[AT1G07400, 17.8 kDa class I heat shock protein (HSP17.8-CI)] |
| AT1G53540 | 260978_at | 16.43 | 17.6 kDa class I small heat shock protein (HSP17.6C-CI) (AA 1-156) |
| AT5G12020 | 250296_at | 13.43 | HSP17.6II (17.6 KDA CLASS II HEAT SHOCK PROTEIN) |
| AT2G29500 | 266294_at | 7.99 | 17.6 kDa class I small heat shock protein (HSP17.6B-CI) |
| AT1G74310 | 260248_at | 7.59 | ATHSP101 (HEAT SHOCK PROTEIN 101); ATP binding / ATPase |
| AT5G52640 | 248332_at | 7.54 | HSP81-1 (HEAT SHOCK PROTEIN 81-1); ATP binding / unfolded protein binding |
| AT5G51440 | 248434_at | 7.34 | 23.5 kDa mitochondrial small heat shock protein (HSP23.5-M) |
| AT1G52560 | 262148_at | 5.68 | 26.5 kDa class I small heat shock protein-like (HSP26.5-P) |
| AT3G12580 | 256245_at | 4.41 | HSP70 (heat shock protein 70); ATP binding |
| AT3G08970 | 258984_at | 3.89 | DNAJ heat shock N-terminal domain-containing protein |
| AT2G20560 | 263374_at | 3.56 | DNAJ heat shock family protein |
| AT1G16030 | 261838_at | 3.05 | HSP70B (heat shock protein 70B); ATP binding |
| AT4G25200 | 254059_at | 2.53 | ATHSP23.6-MITO (MITOCHONDRION-LOCALIZED SMALL HEAT SHOCK PROTEIN 23.6) Arabidopsis thaliana oligonucleotide no |
| ***Cell Wall*** |  |  |  |
| AT4G14130 | 245325_at | 4.01 | XTR7 (XYLOGLUCAN ENDOTRANSGLYCOSYLASE 7) |
| AT3G45970 | 252563_at | 3.93 | ATEXLA1 (ARABIDOPSIS THALIANA EXPANSIN-LIKE A1) |
| AT5G57560 | 247925_at | 3.43 | TCH4 (TOUCH 4), hydrolase acting on glycosyl bonds |
| AT3G55500 | 251791_at | 3.27 | ATEXPA16 (ARABIDOPSIS THALIANA EXPANSIN A16) |
| AT2G47550 | 245151_at | 3.04 | pectinesterase family protein |
| AT2G43870 | 260608_at | 2.81 | polygalacturonase, putative / pectinase, putative |
| AT1G35230 | 259550_at | 2.48 | Encodes arabinogalactan-protein (AGP5) |
| AT4G25810 | 254042_at | 2.34 | XTR6 (XYLOGLUCAN ENDOTRANSGLYCOSYLASE 6) |
| AT1G03870 | 265066_at | 2.29 | fasciclin-like arabinogalactan-protein 9 (Fla9) |
| AT4G30270 | 253666_at | 2.26 | MERI5B hydrolase |
| AT3G27400 | 257735_at | 2.21 | pectate lyase family protein |
| AT1G02640 | 260914_at | 2.20 | BXL2 (BETA-XYLOSIDASE 2) |
| AT2G43050 | 265246_at | 2.19 | pectinesterase family protein |
| AT4G13340 | 254770_at | 2.18 | leucine-rich repeat family protein / extensin family protein similar to LRX2 |
| AT1G10550 | 263207_at | 2.12 | XTH33 (xyloglucan:xyloglucosyl transferase 33) |
| AT4G03210 | 255433_at | 2.07 | XTH9 (XYLOGLUCAN ENDOTRANSGLUCOSYLASE/HYDROLASE 9) |
