# Supplementary material for: Genetic and Genomic Analysis of Rhizoctonia solani Interactions with Arabidopsis; Evidence of Resistance Mediated through NADPH Oxidases
Source: PLoS One. 2013 Feb 25;8(2):e56814. doi: 10.1371/journal.pone.0056814 (PMC3581538; doi:10.1371/journal.pone.0056814)
Supplement: Table S4 — Affymetrix results of selected Arabidopsis genes of seedlings infected with R.solani AG2-1 vs AG8. Genes with higher expression in R. solani AG2-1 infected tissue compared to R. solani AG8infected tissue (adjusted P value<0.05 and a fold change >2.0). (DOCX) [file pone.0056814.s007.docx]

**Table S4**

| **AGI number** | **Probe set** | **fold** | **Gene name** |
| --- | --- | --- | --- |
| ***Cytochrome*** |  |  |  |
| AT5G47990 | 248727_at | 6.98 | CYP705A5 (cytochrome P450, family 705, subfamily A, polypeptide 5); oxygen binding |
| AT5G47990 | 248727_at | 6.98 | CYP705A5 (cytochrome P450, family 705, subfamily A, polypeptide 5); oxygen binding |
| AT5G48000 | 248728_at | 4.01 | CYP708A2 (cytochrome P450, family 708, subfamily A, polypeptide 2); oxygen binding |
| AT5G42590 | 249203_at | 2.96 | CYP71A16 (cytochrome P450, family 71, subfamily A, polypeptide 16); oxygen binding |
| AT5G25120 | 246947_at | 2.22 | CYP71B11 (cytochrome P450, family 71, subfamily B, polypeptide 11); oxygen binding |
| AT3G26290 | 257628_at | 2.05 | CYP71B26 (cytochrome P450, family 71, subfamily B, polypeptide 26); oxygen binding |
| AT3G03470 | 259058_at | 2.03 | CYP89A9 (cytochrome P450, family 87, subfamily A, polypeptide 9); oxygen binding |
| AT2G45560 | 267505_at | 2.03 | CYP76C1 (cytochrome P450, family 76, subfamily C, polypeptide 1); heme binding / iron ion binding / monooxygenase |
| ***GST*** |  |  |  |
| AT1G49860 | 259813_at | 5.44 | ATGSTF14 (Arabidopsis thaliana Glutathione Stransferase (class phi) 14); glutathione transferase |
| AT3G03190 | 258851_at | 2.14 | ATGSTF11 (GLUTATHIONE STRANSFERASE F11); glutathione transferase |
| AT1G17190 | 262516_at | 2.08 | ATGSTU26 (Arabidopsis thaliana Glutathione Stransferase (class tau) 26); glutathione transferase |
| ***Peroxidase*** |  |  |  |
| AT5G64100 | 247297_at | 3.58 | peroxidase, putative |
| AT4G30170 | 253667_at | 2.63 | peroxidase, putative |
| AT2G18980 | 266941_at | 2.43 | peroxidase, putative |
| AT1G49570 | 261606_at | 2.09 | peroxidase, putative |
| AT1G30870 | 265102_at | 2.01 | cationic peroxidase, putative |
| ***FAD-binding*** |  |  |  |
| AT2G34810 | 267425_at | 2.10 | FADbinding domaincontaining protein |
| ***Chitinase*** |  |  |  |
| none |  |  |  |
| ***Pathogenesis*** |  |  |  |
| AT2G19990 | 265586_at | 3.32 | PR1LIKE (PATHOGENESISRELATED PROTEIN1LIKE) |
| AT4G07820 | 255160_at | 2.85 | pathogenesisrelated protein, putative |
| AT2G19970 | 265588_at | 2.54 | pathogenesisrelated protein, putative |
| ***ERF/AP2*** |  |  |  |
| AT2G44940 | 266820_at | 2.03 | AP2 domaincontaining transcription factor TINY, putative |
| ***NAC domain*** | |  |  |
| none |  |  |  |
| ***Response Regulator*** | |  |  |
| AT5G24470 | 249741_at | 2.00 | APRR5 (PSEUDORESPONSE REGULATOR 5); transcription regulator |
| ***WRKY*** |  |  |  |
| none |  |  |  |
| ***heat shock protein*** | |  |  |
| none |  |  |  |
| ***Cell wall*** |  |  |  |
| AT2G45220 | 245148_at | 3.00 | pectinesterase family protein |
| AT5G14650 | 250142_at | 2.77 | polygalacturonase, putative / pectinase, putative |
| AT3G62680 | 251226_at | 2.65 | PRP3; PROLINERICH PROTEIN 3 |
| AT5G40730 | 249375_at | 2.01 | AGP24 (ARABINOGALACTAN PROTEIN 24) |
