# Supplementary material for: Genetic and Genomic Analysis of Rhizoctonia solani Interactions with Arabidopsis; Evidence of Resistance Mediated through NADPH Oxidases
Source: PLoS One. 2013 Feb 25;8(2):e56814. doi: 10.1371/journal.pone.0056814 (PMC3581538; doi:10.1371/journal.pone.0056814)
Supplement: Table S5 — List of relevant oligonucleotides. Primer Sequences (5′ – 3′) used in quantitative RT-PCR and cloning. (DOCX) [file pone.0056814.s008.docx]

**Table S5**

| Name | Oligonucleotide Primer1 | Oligonucleotide Primer1 |
| --- | --- | --- |
| **AG8 rDNA** | AGAGTTGGTTGTAGCTGGTCC | CCGTTGTTGAAACTTAGTATTAGA |
| **At β-tubulin** | ATCACAGCAATACAGAGCCTTAACC | GCTGTTGTTATTGCTCCTCCTGCA |
| **AtrbohD** | CTGGACACGTAAGCTCAGGA | GCCGAGACCTACGAGGAGTA |
| **AtrbohF** | TCACAAATCAACGACGAGAGTT | CCCATCTTCATTCTTGTCCA |
| **Bos1** | GCTGTAGACTAAGGTGGTTA | TTGCACCCGCGTCCTCCAGT |
| **ChitB** | CGGTGGTACTCCTCCTGGACCCA | CGGCGGCACGGTCGGCGTCTGAA |
| **GSTF7** | CCACCTTGCTTTAAGAACAAAGTC | TTGGAGCCAAGGGAGACAAGTTGG |
| **HSP17.4** | TATCAACGGACTTGACCTCC | CTTCAGATAAGCGGTGAGAG |
| **HSP17.6A** | CACACCGTCATTACAAGCCGC | CGAGAAGACTCGTAACAACC |
| **Lox2** | GTTGGATCTTTTATCAACAC | CATACTTAACAACACCAGCT |
| **oxHSP17.4** | GatewayAdapter+  CGAAGAGAGTTCTAGCAAA | GatewayAdapter+  GACTGAGGTCCCTTCACTTA |
| **oxHSP17.6A** | GatewayAdapter+  AAAAGCCAAGAAGCAAGAAAG | GatewayAdapter+  CGAACACCAAGAGGTAGTT |
| **PAD3** | ACTCTGGGAAAACGCAGATGAG | TGATCTCTTTGGCTTCCTCCTG |
| **PDF1.2** | TGTTCTCTTTGCTGCTTTCGACG | GCATGATCCATGTTTGGCTCCT |
| **PR1** | TTCTTCCCTCGAAAGCTCAA | AAGGCCCACCAGAGTGTATG |
| **PR4** | TGCTACATCCAAATCCAAGCCT | CGGCAAGTGTTTAAGGGTGAAG |
| **WRKY33** | GAAAGGGGACAATGAAACAA | GGTTGTGATTACTGCTCTCA |
